# Dielectric Response of ZnO/PMMA Nanocomposites with Atmospheric Pressure Plasma-Modified Surfaces

**DOI:** 10.3390/ma17164063

**Published:** 2024-08-15

**Authors:** Anastasios C. Patsidis, Panagiotis Dimitrakellis, Evangelos Gogolides, Georgios C. Psarras

**Affiliations:** 1 Smart Materials & Nanodielectrics Laboratory, Department of Materials Science, School of Natural Sciences, University of Patras, 26504 Patras, Greece; g.c.psarras@upatras.gr; 2 Institute of Nanoscience and Nanotechnology, NCSR “Demokritos”, Aghia Paraskevi, 15341 Attiki, Greece; p.dimitrakellis@certh.gr (P.D.); e.gogolides@inn.demokritos.gr (E.G.)

**Keywords:** polymer nanocomposites, atmospheric plasma, selective etching, dielectric properties, zinc oxide

## Abstract

In this work, the effect of etching the surface of polymer matrix nanocomposites with atmospheric pressure plasma targeting to achieve enhanced dielectric properties was investigated. Polymer nanocomposites, with varying reinforcing phase content, were modified by atmospheric-pressure plasma resulting in an increase in the surface filler’s concentration. Polymethyl methacrylate (PMMA) matrix nanocomposites reinforced with zinc oxide (ZnO) nanoparticles were prepared and dielectrically studied as a function of the nanoparticle content and the plasma modified surfaces. The electrical response of the composite systems was studied by means of Broadband Dielectric Spectroscopy (BDS) over a wide range of temperatures and frequencies. The dielectric permittivity increased with the embedded phase content and with plasma surface treatment. Energy density followed the same trend as dielectric permittivity, and the plasma-treated nanocomposite with the higher ZnO content exhibited approximately 27% higher energy density compared to the unreinforced matrix.

## 1. Introduction

Dispersing semiconductive nanoinclusions within a polymer matrix results in the development of nanocomposite systems which have been found to exhibit enhanced dielectric responses [1,2,3,4,5]. Polymers are characterized by a low real part of dielectric permittivity and high dielectric breakdown strength. Their permittivity can be significantly increased by integrating ceramic nanoparticles, while the resulting nanocomposites maintain the advantages of the polymeric matrix. Thus, ceramic/polymer nanocomposites can be fabricated easily in various forms and shapes, and exhibit thermomechanical stability and resistance to corrosive environments, at a low cost. Moreover, it has been found that ceramic nanoparticles can prove beneficial in terms of static and dynamic mechanical properties, and in the case of magnetic inclusions and/or ferroelectric filler, magnetic response and multifunctional performance is induced in the nanocomposites [6,7,8,9,10,11]. Nanocomposites of a semiconductive reinforcing phase and an insulating polymer matrix are considered to be suitable systems for electrical energy storage, since the dispersed nanoparticles can act as a distributed network of nanocapacitors [2,3,12,13,14,15]. However, energy can be stored in all three components of the systems, that is the polymer matrix, the ceramic filler and the constituents’ interface [16].

This type of engineering material could prove useful in applications where integrated capacitors are needed, or in cases where structural elements should be able to store and recover energy, while having adjustable electrical or more general physical properties. The electrical response of nanocomposite materials can be tuned suitably by controlling the type and the amount of the reinforcing inclusions. Combining a tunable electrical performance with mechanical rigidity, thermomechanical stability, and ease of processing in tandem with low-cost results in the next generation of engineering materials.

However, there are applications where the final efficiency of a material is not defined completely by the material’s structure and bulk properties, but is also dependent upon its surface properties [17,18,19,20,21,22,23,24,25,26,27,28]. Therefore, a possible modification of the nanocomposite’s surface could prove important for the electrical or other physical properties of the material. Non-thermal plasmas are widely used for the surface treatment of materials (functionalization, etching, and film deposition) without affecting the bulk properties of the material [29,30].

Polymethyl methacrylate (PMMA) is an extensively used engineering polymer. PMMA is produced by in-chain polymerization of the methyl methacrylate group. Its low density, high processability, shape forming, and suitable thermomechanical properties, optical transparency, dielectric properties, and low cost have been exploited in various applications in the fields of electronic devices, optical apparatus, optoelectronic systems, civil and architecture engineering, and elsewhere for aesthetic components. Moreover, PMMA exhibits high biocompatibility, hemocompatibility, and transparency, which made it suitable for various biomedical applications in orthopedics, bone tissue engineering, and dentistry, etc. [31]. PMMA has been also used as the polymeric matrix for the fabrication of polymer matrix nanocomposites (PNCs). The employed reinforcing phases include forms of nano-carbon, metallic nanoparticles such as Ag, synthetic fibers, and various oxides (i.e., ZnO, TiO_2_, and SnO_2_) [32]. Fabricated PMMA nanocomposites are used in optoelectronic devices, biomedical applications, water purification, and energy storage devices [31,33,34,35]. Typical fabrication methods for PNCs are: (a) solution blending or solvent-assisted methods, (b) melt blending, and (c) in situ polymerization [36]. Since PMMA’s repeat unit includes hydrophilic (carbonyl) and hydrophobic (methylene) groups, plasma treatment has been employed to modify its surface properties. Thus, surface adhesion and wettability can be enhanced, and at the same time, friction can be reduced [37]. Recently, it has been reported that plasma treatment of the surface of PMMA or PMMA composites is used for adjusting surface roughness, mechanical properties, and cell-polymer interactions [38,39].

ZnO is a wide band gap n-type semiconductive material, which crystallizes in the hexagonal wurtzite structure. The latter is responsible for its polar structure along the c-axis [40,41]. Nanostructures of ZnO have been reported to have interesting properties such as, non-toxicity, biocompatibility, high transparency, variable conductivity, piezoelectric behavior, magneto-optic response, and chemical sensing. For these reasons, it is used in sensors/actuators, chemical and biochemical sensors, blue lasers, telecommunication, optical wave guide, mechanical and solar energy harvesting, emitting diodes, and biomedical devices [41].

In this study, for the first time, to the best of our knowledge, an investigation was conducted on the effect of the surface texturing of polymer matrix nanocomposites with atmospheric plasma etching on their electrical properties. By these means, the selective removal of a polymer matrix from the top surface is achieved, leading to an increase in the surface concentration of the embedded nanoparticles. The constituent materials of the nanocomposites are polymethyl methacrylate (PMMA) as the matrix and zinc oxide (ZnO) nanoparticles as the reinforcing phase. The dielectric response of the nanocomposites is investigated via broadband dielectric spectroscopy, in a wide frequency and temperature range, prior to and after the plasma treatment. For comparison reasons the electrical properties of the unfilled PMMA were also studied.

## 2. Materials and Methods

In the present study, polymer nanocomposites were fabricated by employing polymethyl methacrylate (PMMA) as the matrix and ZnO nanoparticles as the reinforcing phase. PMMA had MW = 120 kDa, and the diameter of the nanoparticles was less than 100 nm. Both ingredients were purchased from Sigma-Aldrich (St. Louis, MO, USA). PMMA was dissolved in propylene glycol methyl ether acetate (PGMEA) solvent prior to the nanoparticles’ dispersion. ZnO nanoparticles were in the form of spheroids with diameters less than 100 nm, according to the manufacturer. The content of the ZnO nanoparticles was 3 and 6 phr (parts per hundred resin per mass). ZnO/PMMA films were spin-coated on 0.8 mm thick PMMA plates. The width of the nanocomposite films was approximately 3.5 μm. For comparison reasons an unreinforced specimen was also prepared.

The etching or nanotexturing of the specimens was conducted via atmospheric-pressure plasma in a dielectric barrier discharge (DBD) reactor operating at 13.56 MHz, which is suitable for the large-area, homogeneous modification of plane materials under ambient conditions. Helium and oxygen (Air Liquide) were introduced directly into the plasma zone through the showerhead RF electrode at a total mass flow rate of 5 standard liters per minute (slm) and a fixed gas composition (0.6% O_2_ in He). The employed RF power was constant at 120 W and all specimens were treated for up to six minutes, at successive plasma on (5 s) and plasma off (15 s) periods. By these means, the heating of the surfaces was avoided. Detailed descriptions of the specimens’ preparation and plasma etching can be found in [42].

Dielectric measurements were conducted via Broadband Dielectric Spectroscopy (BDS) in the frequency range from 10^−1^ to 10^7^ Hz via a frequency response analyzer (Alpha-N Analyzer, Novocontrol Technologies, Montabaur, Germany). Specimens were subjected to isothermal frequency scans, while arranged in a dielectric cell (BDS 1200, Novocontrol Technologies) in a parallel plate configuration. Electrodes were gold plated and the applied V_rms_ was constant at 1 V. Temperature was controlled via Novotherm apparatus (Novocontrol Technologies) in the range 30 to 160 °C, with a 10 °C step between isothermal scans. Data recording was carried out in real time via WinDeta software, also supplied by Novocontrol Technologies. BDS allows the accurate recording of the variations of the real and imaginary part of dielectric permittivity as a function of frequency and temperature. Thus, other electrical quantities, such as the real and imaginary parts of the electric modulus, loss tangent delta, and AC conductivity, can be easily determined.

## 3. Results

**Figure 1:**
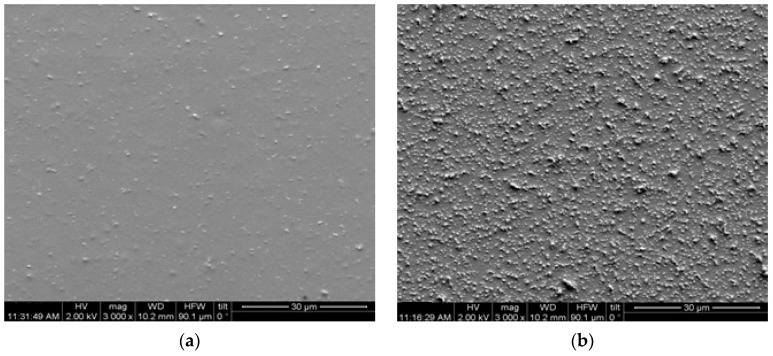
SEM images from the nanocomposite with 3 phr ZnO: (**a**) prior to applying plasma treatment and (**b**) after the application of plasma for 6 min (magnification: 3000×).

The effect of plasma texturing upon the surface of the examined nanocomposites was demonstrated via Scanning Electron Microscopy (SEM) images. Figure 1 presents representative SEM images from the specimen with 3 phr ZnO prior the application of plasma (t = 0 min) and after plasma treatment for t = 6 min, at the same magnification. The modification of the specimens’ surface topography is apparent. In Figure 1a the presence of ZnO nanoparticles can be identified, although most of them are covered by the polymer and only a small percentage lie at the surface. The situation becomes different after the plasma treatment and numerous nanoparticles can be clearly seen at the etched surface (Figure 1b).

**Figure 2:**
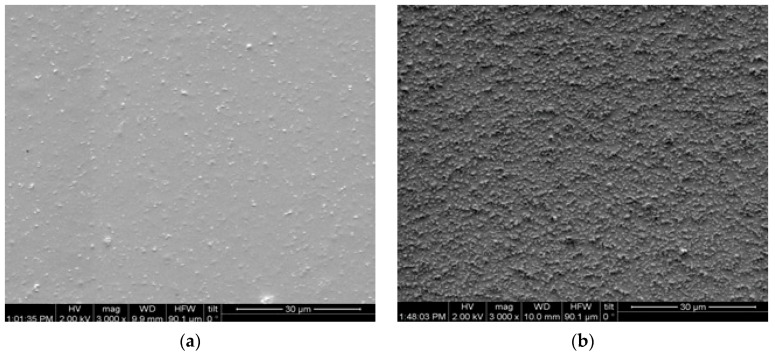
SEM images from the nanocomposite with 6 phr ZnO: (**a**) prior to applying plasma treatment and (**b**) after the application of plasma for 6 min (magnification 3000×).

In Figure 2, the corresponding images of the nanocomposite with 6 phr ZnO nanoparticles are shown. The images confirm the revealing of nanoparticles at the modified surface, as well as the higher filler concentration.

**Figure 3:**
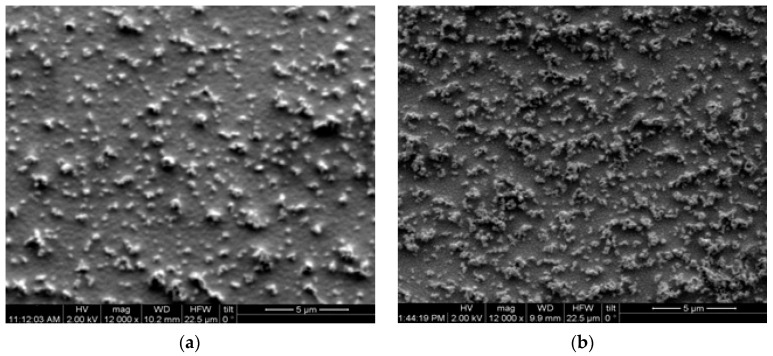
SEM images from the nanocomposite with: (**a**) 3 phr ZnO and (**b**) 6 phr ZnO, after the application of plasma for 6 min, at 12,000×.

The resulting surface modification with plasma treatment is highlighted in the images in Figure 3, where the surfaces of the nanocomposites with 3 and 6 phr ZnO nanoparticles are shown at a higher magnification.

**Figure 4:**
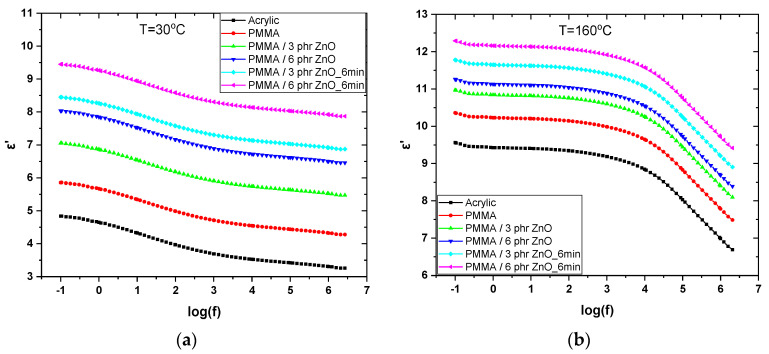
Real part of dielectric permittivity as a function of frequency at: (**a**) 30 °C and (**b**) 160 °C for all the studied systems.

The dielectric response of the studied nanocomposites is mainly expressed via the real part of dielectric permittivity (*ε*′), which historically is known as a dielectric constant. Figure 4 depicts the variation of *ε*′ with frequency at the lower (30 °C, Figure 4a) and at the higher (160 °C, Figure 4b) applied temperature. In these plots, comparative spectra of the unreinforced PMMA and the nanocomposites with 3 and 6 phr ZnO nanoparticles, prior to and after being subjected to plasma treatment are given. Since the nanocomposites have been developed on an acrylic wafer, the dielectric response of the substrate is also presented in Figure 4.

As expected, the nanocomposites exhibit higher values of *ε*′ than unreinforced PMMA in the whole frequency range, at both temperatures. The real part of dielectric permittivity of ceramic ZnO nanoparticles is higher than the corresponding one for the polymer matrix. Thus, the increment of *ε*′ with filler content is reasonable. In addition, the electrical heterogeneity between the constituents of the nanocomposites gives rise to interfacial polarization (also referred to as the Maxwell–Wagner–Sillars/MWS phenomenon). The employed ceramic particles were on a nanometric scale (<100 nm), and for this reason an extensive interface between matrix and filler was developed in the nanocomposites. At this interface, unbounded charges are trapped, forming dipoles which have the size of the nanoparticles or of small clusters. Since the size of the dipoles at the interface is larger (much larger than the atomic scale) than the relative of other permanent or induced dipoles in the systems, they are characterized by enhanced inertia.

Each spectrum attains higher values in the low frequency region, which diminish as the frequency increases. This gradual decrease signifies the failure of induced and permanent dipoles to follow the increasing polar alternation of the field. Thus, the dipoles gradually fail to be aligned with the field, resulting in a lower level of polarization and lower values of the real part of the dielectric permittivity. This behavior is indicative of the presence of dielectric relaxations [1,9,10,11,43]. In addition, temperature acts as an extra factor facilitating the dipole alignment. Increases in temperature result in the agitation of the dipoles and charges, which become orientated by the field, which is expressed via the enhanced values of *ε*′. The effect of frequency and temperature is common for all dielectric spectra recorded.

Interestingly, the plasma-treated nanocomposites exhibit the highest values of *ε*′. It is worth noting that the plasma specimen treated for 6 min with 3 phr ZnO, has higher values for the real part of dielectric permittivity than the untreated specimen with 6 phr ZnO (Figure 4). The surface modification of the nanocomposites with plasma removes the polymeric matrix, thus altering the concentration of the ZnO nanoparticles in the specimens. Apparently, the plasma modified system with 6 phr ZnO content, displays the highest values for *ε*′ in the whole frequency and temperature range.

**Figure 5:**
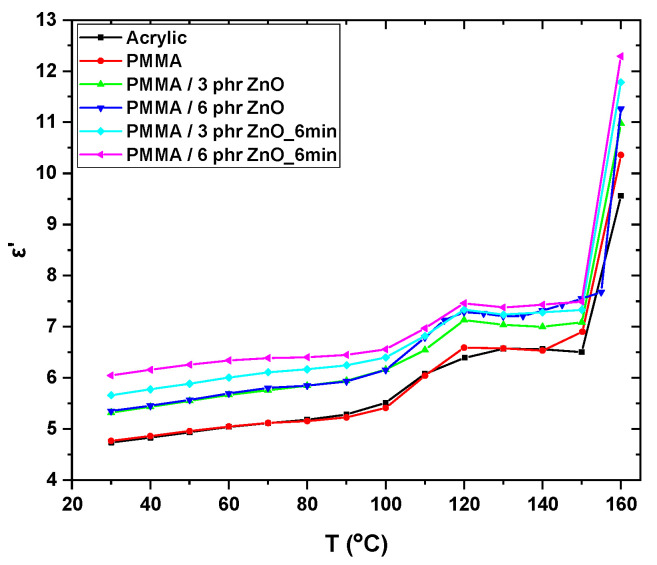
Real part of dielectric permittivity versus temperature, at 0.1 Hz, for all studied systems.

An analogous picture is shown in Figure 5, where *ε*′ varies with temperature at 0.1 Hz. Reinforced systems exhibit higher values of permittivity, while the spectra of acrylic substrate and unreinforced PMMM have an almost identical response due to the chemical proximity of their structures. Again, the plasma modified systems attain the highest values, as noted in Figure 4. In all cases, in the temperature region between approximately 100 and 120 °C, an increase in *ε*′ values is recorded, which is followed by a plateau. This enhancement in permittivity values indicates an increase in the achieved polarization because of the orientation of polar molecular parts and polar segments with the applied field. This indicates that the glass to rubber transition of PMMA occurs in this region. The latter is in accordance with previous studies in the literature [44,45,46,47]. The consequent plateau, between 120 and 150 °C, confirms the completion of the glass to rubber transition process (α-relaxation). However, the values of the real part of dielectric permittivity increase at the highest measured temperature. In the nanocomposite systems, this rise of *ε*′ could be attributed, reasonably, to the interfacial polarization (MWS effect) because of the accumulation of free charges at the nanocomposite interface [1,48]. PMMA and acrylic substrate are amorphous polymers and in their unreinforced form should not exhibit interfacial polarization. However, Figure 5 shows that although they are at a lower level, they follow the same trend as the nanocomposites at the higher temperature edge. It should be noted that in amorphous unreinforced polymers, interfacial polarization has been observed and reported due to the presence of additives and plasticizers [1,49,50].

## 4. Discussion

PMMA is a transparent amorphous thermoplastic polymer with sufficient mechanical properties, environmental safety, reduced cost, low thermal and electrical conductivity, feasible formation, and low cost [44,45,47,51]. Among other applications, it is considered a suitable matrix for the fabrication of micro- and nanocomposites [52,53]. The dielectric spectrum of PMMA includes relaxations related to its molecular structure. With increasing temperature, at a constant frequency, they are designated as γ-, β-, and α-relaxation. α-Relaxation corresponds to the glass to rubber transition and is characterized by longer relaxation times, β-relaxation is assigned to local rotations of the polar ester side-group, and the weak γ-relaxation to isolated rotations of the methyl groups of the main chain [44,45,46]. The dynamics of α- and β-relaxations differ, and thus at high temperatures these processes merge into a single mode referred to as αβ-relaxation, in which the contribution of each mode is difficult to determine. In addition to these three relaxations, interfacial polarization and conductivity relaxation have also been reported in unreinforced PMMA [47].

**Figure 6:**
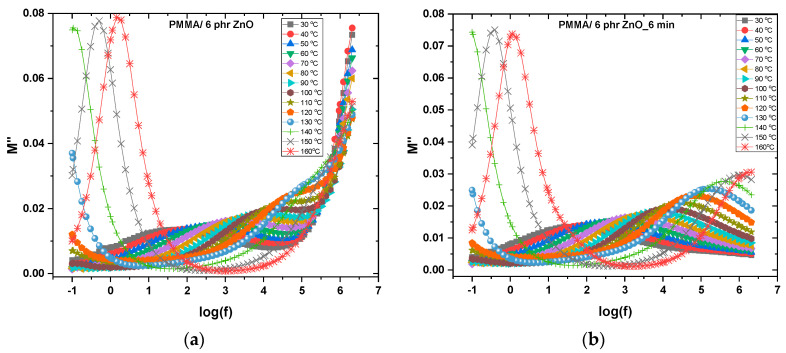
Electric modulus loss index as a function of frequency at various temperatures for PMMA/6 phr ZnO nanocomposite (**a**) prior to and (**b**) after plasma treatment.

Dielectric relaxation can be more easily detected and studied via dielectric loss spectra. The latter are expressed in different formalisms, namely, the imaginary part of permittivity (*ε*″), the electric modulus loss index (*M*″), and the loss tangent delta (tan*δ*) [1,50]. Figure 6 presents the variation of the imaginary part of electric modulus (loss index *M*″) versus frequency for the nanocomposite with 6 phr ZnO prior to and after plasma treatment for 6 min.

In both plots, the dielectric spectra clearly imply the presence of two relaxation processes. A relatively weaker and broader relaxation with its peaks covering a wide frequency range (from 10 to 10^6^ Hz) and a sharper one at low frequencies. The second one is attributed to interfacial polarization with the possible contribution of conductivity relaxation. As already stated, interfacial polarization (MWS effect) arises from trapping at the interface of mobile charges. These charges form dipoles, but in a trapping/detrapping procedure could also migrate though the interface, thus contributing to conductivity. The theoretical description of interfacial polarization includes two terms, a dipolar one and a term for conductivity [1,48,50]. The predominant contribution each time depends on the specific constituents, the filler content, the temperature, and the frequency of the field. The first process, the one represented by broad peaks, which covers a temperature range from 30 to 160 °C, is recorded in the frequency and temperature region where α- and β-relaxations are expected. Accounting for its broadness and range, it is assigned to the merge process (αβ-relaxation) of α- and β-relaxations. In Figure 6a, at the high frequency edge, the spectra shows a sharp increase, which is absent in the case of the plasma-treated nanocomposite. At the high frequency edge, the polar moieties which participate in a dielectric relaxation process, characterized by a very small relaxation time, should be small and very light. Hence, it is reasonable to suggest that the participating dipoles in this “half-recorded” process are the remaining polar groups from the employed solvent in the specimens’ fabrication procedure. The spectrum of the plasma treated nanocomposite does not contain this “tail”, since the solvent’s polar group was removed during treatment.

**Figure 7:**
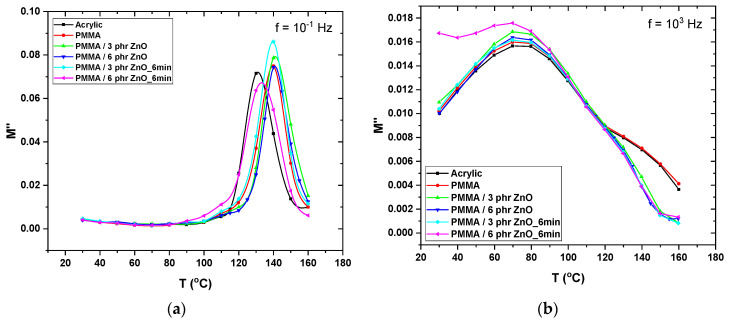
Electric modulus loss index as a function of temperature at: (**a**) 10^−1^ Hz and (**b**) 10^3^ Hz for all the studied systems.

In Figure 7, comparative plots of the electric modulus loss index as a function of temperature, at 0.1 Hz (Figure 7a) and 1 kHz (Figure 7b), are shown for all studied systems.

In Figure 7a, the loss peak of interfacial polarization is clearly formed for all systems in the temperature range of 120 and 160 °C. A slight shift of the peak position indicates a variation in the relaxation times. In the same plot, a small hump is observed between 100 and 120 °C. The latter is suppressed due to the relatively intense peak of interfacial polarization. In Figure 7b, the frequency–temperature superposition shifts the peak of interfacial polarization out of the window of observation. The recorded peaks are broad, and their intensity corresponds to the αβ-relaxation mentioned previously. The hump on the right side of the peaks signifies the presence of α-relaxation. The dynamics of the α-process in unreinforced PMMA and nanocomposites are different, because of the interaction between macromolecules and nanoparticles. This difference is reflected by a more merged peak in the case of nanocomposites.

**Figure 8:**
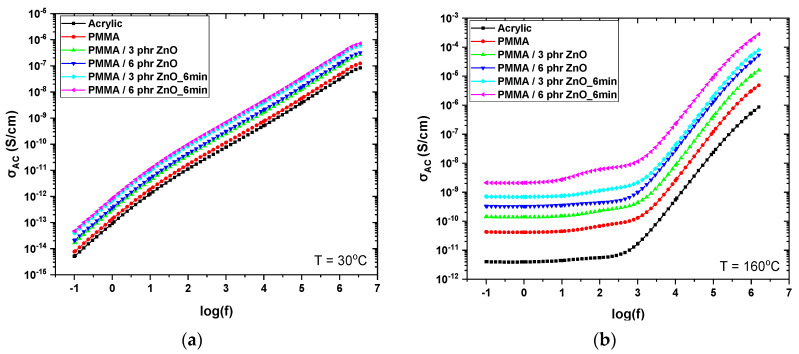
AC conductivity as a function of frequency at: (**a**) 30 °C and (**b**) 160 °C for all the studied systems.

AC conductivity is a physical quantity summing all dissipative processes including an actual ohmic conductivity, caused by the migration of charge carriers through interface or at isolated semiconductive nanoparticles and the frequency dependent dipolar dispersion [54,55]. AC conductivity can be calculated by employing Equation (1) [54]:

(1) $$\sigma_{AC}=\varepsilon_{0}(2\pi f)\varepsilon^{\prime \prime }$$

where *ε*_0_ is the permittivity of vacuum, *f* the frequency of the applied field, and *ε*″ the imaginary part of dielectric permittivity. The evaluated spectra of AC conductivity at 30 and 160 °C for all studied systems are given in Figure 8. Nanocomposites exhibit higher values of *σ_AC_* in the whole frequency range at both temperatures, because of the higher conductivity of the ZnO nanoparticles with respect to the PMMA matrix. In addition, the plasma-treated nanocomposites acquire higher values than the untreated ones due to the enhanced concentration and volume fraction of nanoparticles. However, the dielectric nature of all examined systems is obvious, and the AC conductivity values remain low.

The frequency and temperature dependence of the *σ_AC_* curves is characteristic for dielectric materials [1,50]. At low temperatures (Figure 8a), AC conductivity values display proximity for all systems and only a slight increase is observed with the ZnO content. The values increase with frequency, as predicted by Equation (1), owing to the alternation of the field force charge carriers, which move between nearby sites, thus participating in a forward and backwards motion. The effect of filler content and plasma treatment becomes more pronounced at high temperatures and low frequencies (Figure 8b). In the low frequency region, the slow alternation of the field gives sufficient time to charge carriers to migrate over longer distances within the systems, while the increased temperature provides thermal agitation to carriers. By these means, AC conductivity forms a plateau in the low frequency region approaching its DC value, while after a critical frequency *σ_AC_* follows an exponential dependence on frequency. The recorded behavior of *σ_AC_* is considered typical of hopping conduction [1,50,55]. The obtained results are in accordance with the so called “AC conductivity universality law”, which is expressed via Equation (2) [56]:

(2) $$\sigma_{AC}\left(2\pi f\right)=\sigma_{DC}+A{\left(2\pi f\right)}^{s}$$

where *σ_DC_* is the limiting value of AC conductivity when frequency tends toward zero and the *A* and *s* parameters are dependent on the temperature and the constituent materials of the system [57,58].

**Figure 9:**
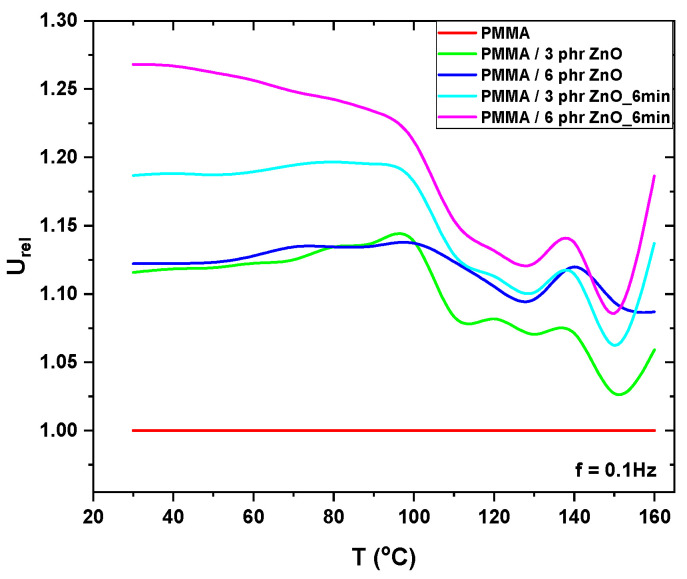
Relative energy density versus temperature, at 0.1 Hz, for all studied systems.

It is well known that dielectric materials can be used as capacitive energy storing devices. Furthermore, nanodispersions in composite materials can act as a distributed network in which electric energy can be stored and recovered [2,12,13,14,15]. The ability of dielectric materials to store energy is expressed by the energy density, which for linear dielectrics (low applied fields) is defined via Equation (3) [2]:

(3) $$U=\frac{1}{2}\varepsilon_{0}\varepsilon^{\prime }E^{2}$$

where *ε*′ is the real part of dielectric permittivity and *E* the intensity of the electric field. Although the energy density is strongly dependent on the applied field, the only material property included in Equation (3) is *ε*′. Therefore, from a material science point of view, the ability to store energy per volume increases with the real part of dielectric permittivity. In the case of reinforced systems, the effect of strengthening the energy storage ability by the presence of filler can be evaluated by employing the relative energy density, which is defined according to Equation (4) [16]:

(4) $$U_{rel}=\frac{U_{comp}}{U_{matrix}}$$

where *U_comp_* is the energy density of the composite material and *U_matrix_* is that of the unreinforced matrix. Figure 9 presents the relative energy density curves of the studied nanocomposites, taking as base the unreinforced PMMA (value 1), versus temperature at 0.1 Hz. The found trends of *U_rel_* are in accordance with the variation of *ε*′ and in the low frequency zone, the increment of energy density for the plasma treated 6 phr ZnO/PMMA nanocomposite, relatively to unreinforced PMMA, is approximately 27%.

In the present study, it was shown that the activation of PNC surfaces via atmospheric plasma results in systems in a higher real part of dielectric permittivity, at low filler content, and at the same time, the ability of storing electrical energy increases. In a future study it could be interesting to investigate the piezoelectric properties of untreated and treated plasma PMMA/ZnO nanocomposites. Recalling the biocompatible properties of both constituents (PMMA and ZnO) and the effect of piezoelectric properties upon bone generation [59], it could be very interesting to examine the effect of the plasma modification of PMMA/ZnO implants.

## 5. Conclusions

In this work, PMMA/ZnO nanocomposites were prepared at two different filler concentrations via spin coating on PMMA plates and subsequently treated with atmospheric plasma. The effect of plasma upon the surface morphology of the nanocomposites was studied via SEM images. The dielectric response of unreinforced PMMA nanocomposites before and after plasma treatment was studied by means of broadband dielectric spectroscopy, with respect to the frequency of the field and the applied temperature. Nanocomposites exhibit higher values of the real part of dielectric permittivity than the unreinforced PMMA, while plasma-treated nanocomposites attain even higher values. The removal of polymer from the specimens’ surface in the treated systems results in increasing the filler concentration and thus, -ε′. In the loss spectra of the examined systems, two relaxation processes are clearly recorded, and are attributed to interfacial polarization and to the merge αβ-process, which is the result of the superposition of α-relaxation (glass to rubber transition) and β-relaxation (reorientation of ester side-groups). Isochronal spectra, at 1 kHz, gave the opportunity for an indirect identification of α-relaxation. The AC conductivity remained low in the whole frequency and temperature range of measurements. Conductivity increases with ZnO content and plasma treatment were in accordance with the “AC universality law”. Temperature is more effective in the low frequency region, where AC conductivity values remain almost constant, tending to acquire their DC value. Above a critical frequency, *σ_AC_* varies exponentially with frequency. Finally, energy density increases with filler content and plasma treatment, revealing the ability of nanocomposites to be used as capacitive energy storing devices. Optimum performance was demonstrated by the plasma treated 6 phr ZnO/PMMA nanocomposite, which exhibited about 27% higher energy storage ability with respect to the unreinforced PMMA. The obtained results denote the possible exploitation of plasma-treated PMMA/ZnO nanocomposites in the field of electronics and energy storing devices, at low filler content.

## Data Availability

Data are available upon request.
